# Supplementary material for: Machine Learning of Hierarchical Clustering to Segment 2D and 3D Images
Source: PLoS One. 2013 Aug 20;8(8):e71715. doi: 10.1371/journal.pone.0071715 (PMC3748125; doi:10.1371/journal.pone.0071715)
Supplement: Figure S1 — Illustration of the VI breakdown plot. (A) The VI breakdown plot shows the conditional entropy of each segment against the segment size (as a proportion of image size). Since the VI is the sum of the products of these two numbers, the hyperbolae are isoclines of contribution to the final VI. It is instantly obvious that the VI is dominated by oversegmentation errors. (B)–(C) Looking at the points with the highest contribution, we can examine the kinds of errors we are making, to improve our algorithm in the future. In this case, segment 18 from the gold standard is split at a “pinch” in the neuronal process, and this is a typical error among the others examined. (It is necessary to look at several planes to determine that the two segments should be connected). (PDF) [file pone.0071715.s001.pdf]

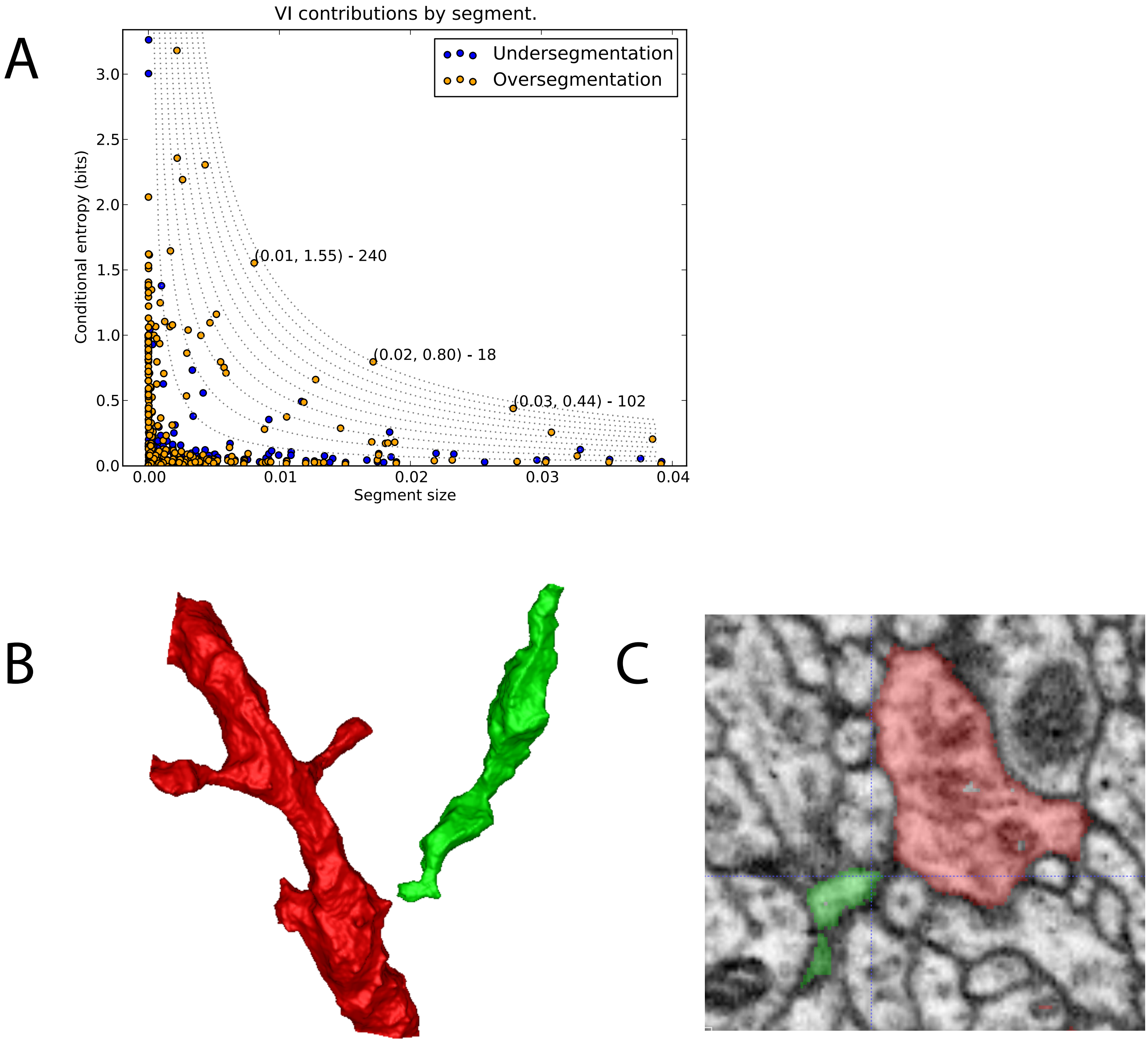

VI breakdown plot. (A) The VI breakdown plot shows the conditional entropy of each segment against the segment size (as a proportion of image size). Since the VI is the sum of the products of these two numbers, the hyperbolae are isoclines of contribution to the final VI. It is instantly obvious that the VI is dominated by oversegmentation errors. (B)-(C) Looking at the points with the highest contribution, we can examine the kinds of errors we are making, to improve our algorithm in the future. In this case, segment 18 from the gold standard is split at a “pinch” in the neuronal process, and this is a typical error among the others examined. (It is necessary to look at several planes to determine that the two segments should be connected.)
