# Supplementary figures and images for: Machine Learning of Hierarchical Clustering to Segment 2D and 3D Images
Source: PLoS One. 2013 Aug 20;8(8):e71715. doi: 10.1371/journal.pone.0071715 (PMC3748125; doi:10.1371/journal.pone.0071715)

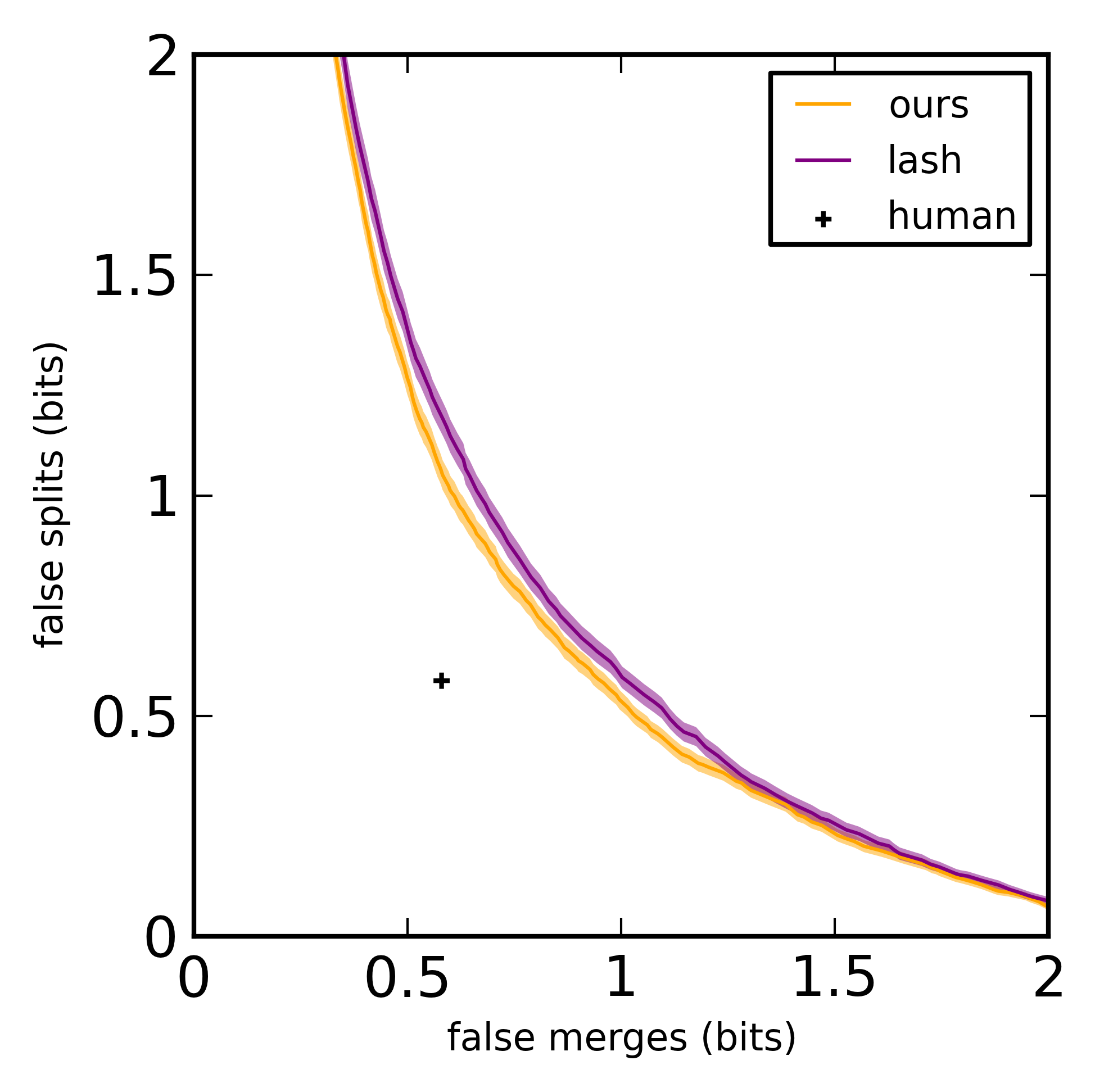

Supplement: Figure S2 — LASH vs GALA performance on natural image data. Note that this plot shows the performance of our own implementation of the LASH learning protocol, using our own features. (TIF) [file pone.0071715.s002.tif]

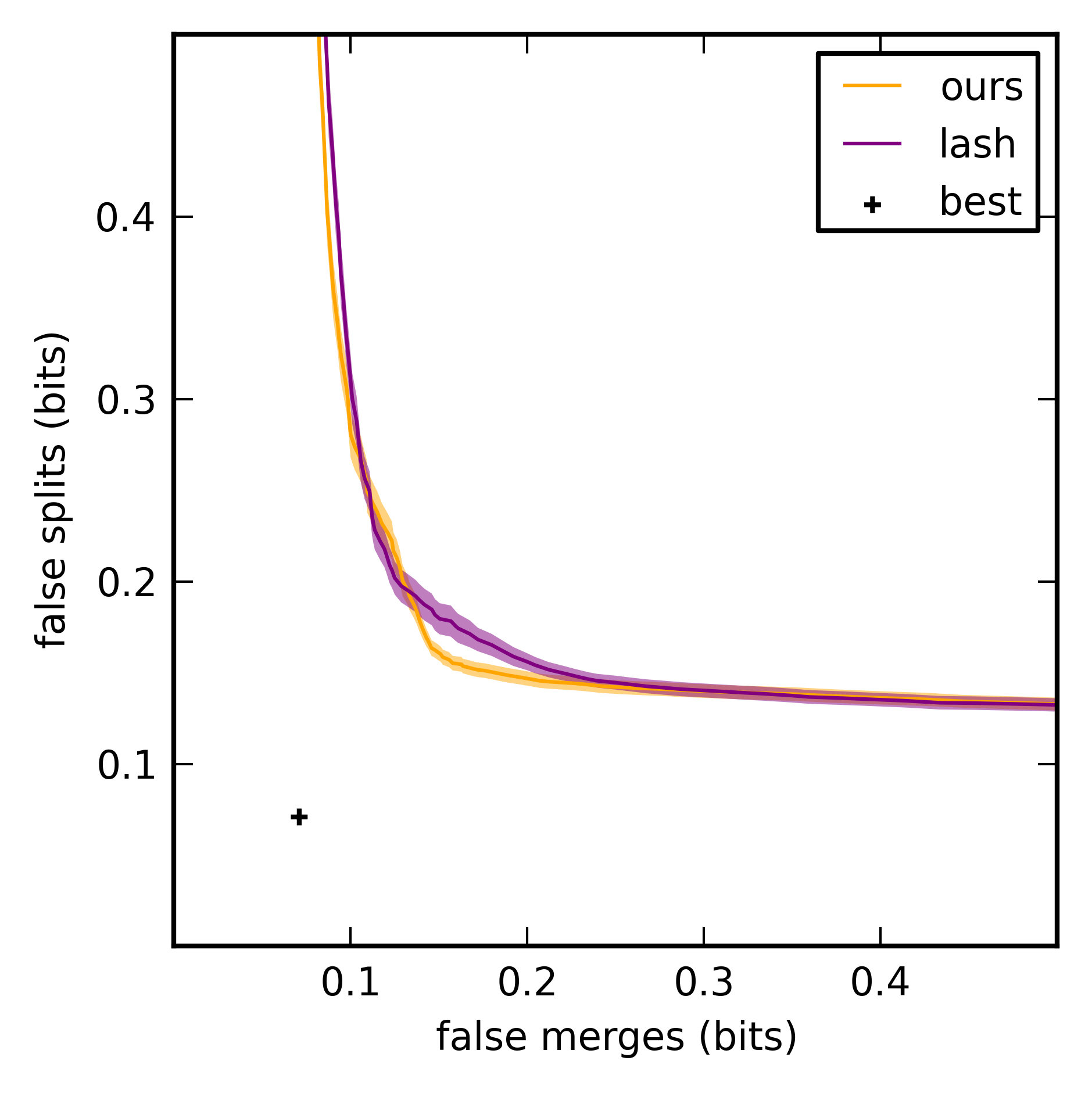

Supplement: Figure S3 — LASH vs GALA performance on our EM dataset. Note that this plot shows the performance of our own implementation of the LASH learning protocol, using our own features. (TIF) [file pone.0071715.s003.tif]
